# The differences in trigeminal system functional state in chronic and frequent migraine patients

**DOI:** 10.1186/1129-2377-14-S1-P125

**Published:** 2013-02-21

**Authors:** G Pavlic, S Odobescu, I Moldovanu, V Lacusta, L Rotaru, G Corcea

**Affiliations:** 1 Institute of Neurology and Neurosurgery, Moldova, Republic of; 2 State Medical and Pharmaceutical University, Moldova, Republic of

## Introduction

Electrophysiological tools like trigeminal somato-sensory evoked potentials (TSSEP) are used for evaluation of trigeminal system (TS) functional state. The activity of the later is an important factor in migraine pathogenesis. The study aim was to analyze the TS functional state in chronic and frequent migraine patients in relation with age during the migraine attack (critic period) and in the interictal period on lateralized stimulation (left/right).

## Results

We have included 170 women with migraine (according to ICHD-IIR, 2006 criteria), between 15-55 years, 109-chronic migraine (CM) and 61-frequent migraine (FM), mean age 38.3±10.7 and 36.5±9.1 years, respectively. In CM vs. FM patients aged 15-25 years a statistic significant reduction in wave latencies N15 (15.4±0.47 vs. 17.67±0.42, p<0.01), P22 (22.24±0.69 vs. 25.11±0.73, p<0.05), interval N6-P9 (3.56±0.29 vs. 5,11±0.56, p<0.05) on the left side and of intervals N6-P9 (3.28±0.40 vs. 5.36±0.54, p<0.05) and N6-P22 (16.76±0.52 vs. 19.18±0.80, p<0.05) on the right was observed, but not in other age groups. Reduced latency and shorter conduction time at nuclear–thalamic level (interval N6-P9) might reflect an increased excitability of thalamic and cortical structures (P22) of trigeminal sensory complex in young patients. There were no differences in TSSEP in CM vs. FM during a migraine attack.

## Conclusions

Functional disturbances in trigeminal system in chronic migraine vs. frequent migraine might be seen in young patients based on latencies of TSSEP and on central time of the somatosensory trigeminal transmission. These changes might reflect a different activity of brainstem-diencephalic (thalamic) structures.
